# Optimisation of Solidification Structure and Properties of Hypoeutectic Chromium Cast Iron

**DOI:** 10.3390/ma15186243

**Published:** 2022-09-08

**Authors:** Dorota Siekaniec, Dariusz Kopyciński, Edward Tyrała, Edward Guzik, Andrzej Szczęsny

**Affiliations:** Faculty of Foundry Engineering, AGH University of Science and Technology, al. A. Mickiewicza 30, 30-059 Kraków, Poland

**Keywords:** chromium cast iron, chromium carbides, modification, inoculants, dilatometric tests, heat treatment, impact strength, abrasive wear, hot cracks

## Abstract

This paper presents a comprehensive approach to optimising the structure and properties of chromium cast iron that is intended for use in the production of castings that operate under abrasive-wear conditions. In the study, chromium cast iron was inoculated to reduce the grain size in the solidification structure. The finer-grained structure of the casting has a positive effect on its mechanical properties. A number of inoculants have been used that allow the elimination of many types of casting defects: hot cracks and porosities that often occur during the production of chromium cast iron castings. Another advantage of the developed inoculation procedure is the resulting increase in the toughness of chromium cast iron. It should be emphasised that this cast iron does not have a high impact strength in its as-cast condition due to the formation of chromium carbides in the structure. This work also proposes a specially designed heat treatment for inoculated cast iron. The parameters of the applied heat treatment were determined on the basis of dilatometric tests. The visible deviation on a dilatogram at a temperature of about 600 °C is the result of a partial martensitic transformation in the area of grain boundaries. Therefore, the increase in abrasion resistance chromium cast iron is mainly due to the appearance of martensite. The microstructure of the investigated cast iron is particularly desirable in the case of alloys that work with lubrication. The microcavities that are formed by the abrasion of the softer phase constitute natural grease, which reduces abrasive wear. Under the influence of heat treatment, only a part of austenite located near the carbides is destabilized and transformed into martensite. Therefore, this phase of composition formation provided much greater resistance to abrasive wear and hardness.

## 1. Introduction

**Figure 1:**
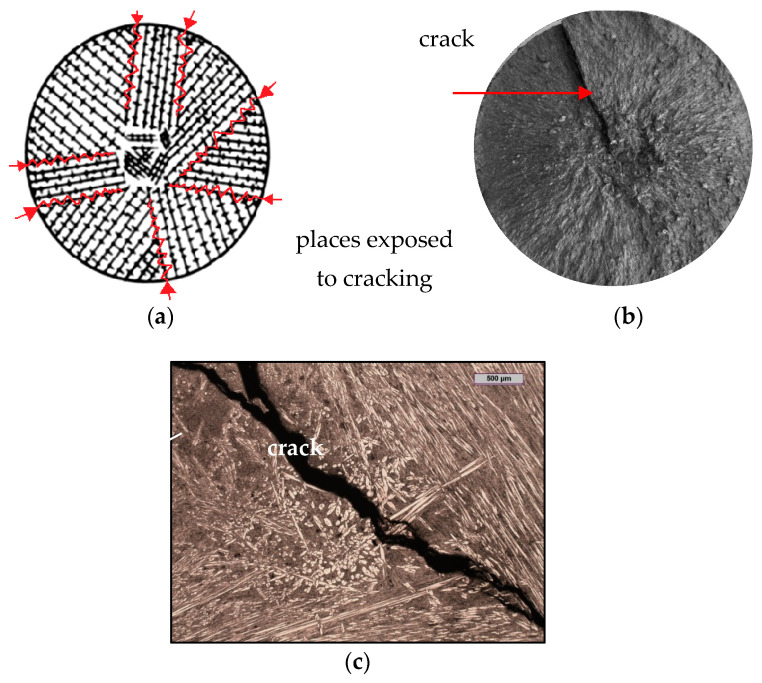
Hot crack propagation in white cast iron with directional of coarse-grained structure: (**a**) scheme of crack formation at grain boundaries [1,2]; (**b**) fracture of chromium cast iron casting with visible crack; (**c**) microstructure of chromium cast iron casting with visible crack (own research).

White cast iron (including chromium cast iron) has a tendency to crystallise directionally [1] (types I and II) and to grow a coarse-grained structure. Therefore, white iron castings with a columnar structure have poorer strength properties (impact and bending strengths). In contrast, crack propagation occurs along grain boundaries [2] (as is suggested in the sample that is shown in Figure 1). These types of cracks occur frequently and are a significant problem in the production of chromium iron castings.

The change in the physicochemical state of the molten metal clearly affects the method of crystallisation. Hence, the conclusion is that the physicochemical state of the molten metal must be changed accordingly to obtain a microstructure of types V and VI morphologies in a white iron casting. The determination of the morphology of a given type can be found in [1]. In this work, one can find a defined series of types of the dendritic crystallisation of primary austenite in white cast iron: type I relates to directional crystallisation (during which the dendrite bundles extend to the axis of the sample); types II, III, and IV—Directional-volume crystallisation (during which the dendrite bundles do not extend to the sample axis); type V—Bulk crystallisation (during which dendrites grow in bundles on the cross-section); VI—Volumetric crystallisation (during which single dendrites grow across the entire cross-section).

**Figure 2:**
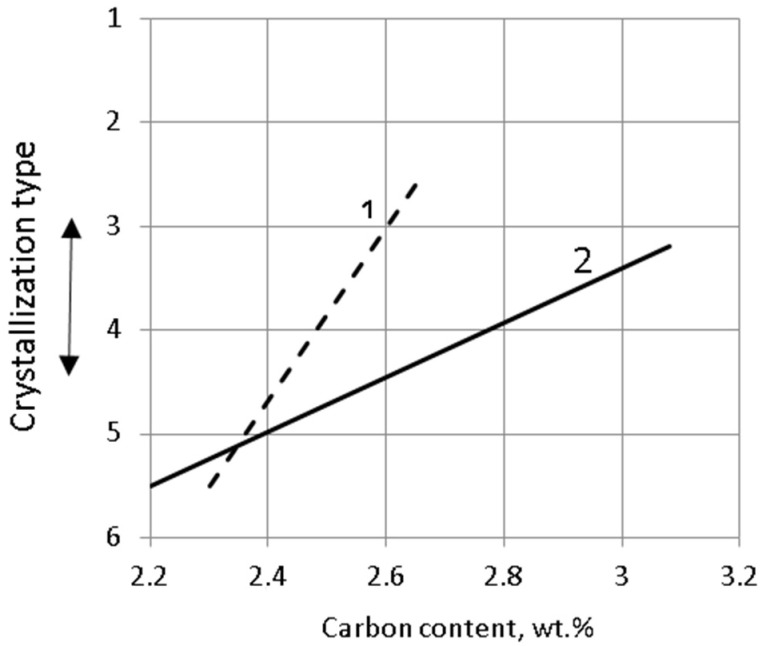
Influence of carbon in molten metal on type of dendritic crystallisation during production of white iron castings: (1) melt in which metal charge contains 20% pig iron and 80% steel scrap; (2) melt in which metal charge consists of 25% pig iron and 75% steel scrap [2].

**Figure 3:**
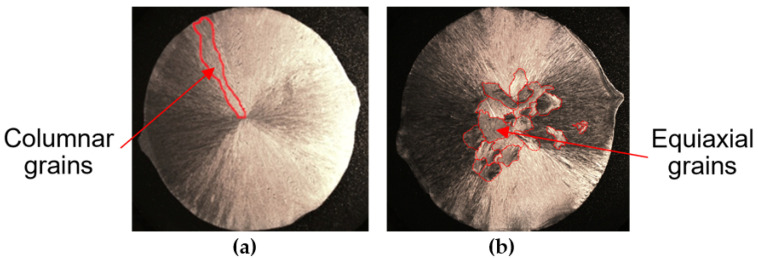
Macrostructure of as-cast high-chromium cast iron: (**a**) before inoculation; (**b**) after inoculation (with marked equiaxial grains) [21].

Such an optimal metal condition can be achieved by the following metallurgical procedures: inoculating the cast, increasing the carbon content, increasing the casting temperature, selecting the appropriate metal charge, increasing the superheat temperature, or shortening the holding time of the molten metal. Figure 2 shows the effect of the metal charge on the type [2] of crystallisation. The graph shows the dependence of the carbon that is contained in the molten metal in correlation with the type of crystallisation according to the series of types presented above. It can be seen from the diagram in Figure 2 that, for the same carbon content of 2.6%, the casting that was obtained from Melt 2 with more pig iron tended to crystallise in a volumetric manner, and the cast that was obtained from Melt 1 crystallised in a directional manner. As a result of increasing the number of nucleation sites, the inoculation procedure also changes the type of crystallisation toward volume crystallisation. There are works that are related to the influence of titanium, vanadium, boron, bismuth [3,4,5,6,7,8,9,10,11,12,13,14], niobium [11,15], tungsten [16,17,18], and molybdenum [19,20] on the structure and properties of chromium cast iron; The chromium cast iron inoculation can be explained by the example shown in Figure 3 [21]. In this study, the reference and inoculated chromium cast iron was poured into a metal mold to produce cast ingots.

If the cast iron is not inoculated, equiaxial grains are not visible on the metallographic specimen (Figure 3a). In this case, metal crystallises (directional) from the side to center of the metal mold. The inoculated chromium cast iron crystallises with the separation of equiaxial grains in the center of the cast ingots (Figure 3b).

In this publication, the influence of various inoculants on the microstructure of high-chromium cast iron and, thus, on the strength properties of castings that are made of this type of iron, has been proven. As can be seen in Figure 3, the biggest problem in the production of chromium cast iron is the coarse-grained structure that is shaped in a directional way from the walls of the casting mould. Thus, it encounters another problem that is related to the tendency toward hot cracking and the appearance of porosity in the castings [22].

In addition, there may also be problems with low hardness and toughness values; hence, this paper will show an example of improving the performance properties of chromium cast iron by using the molten metal inoculation process and a well-designed heat treatment. The presented work is a continuation of the research that was presented in [21,22,23,24].

The properties of chromium cast iron can also be controlled by heat treatment. This, however, cannot eliminate hot cracks during the production of castings. There is much information on this subject in the literature [25,26,27]. Chromium cast irons after heat treatment may have a martensitic matrix with residual austenite. It is generally known that above a content of 18–20% Cr, austenite decomposes into chromium ferrite and carbides. When the castings are to be quenched in order to give them a martensitic matrix, a low Cr/C ratio should be kept to obtain the highest possible hardness of the martensite. The martensitic structure is obtained by quenching in air at a temperature of 900–1000 °C.

Such a temperature makes it possible to dissolve some of the carbides, thus enriching the matrix with chromium and carbon. The increase in the carbon content in the martensite increases its hardness up to as much as 65 HRC. Hardening also has a positive effect on the hardness of cast iron because the high cooling rate reduces the precipitation of the carbides. The most preferred form of carbides are fine-grained precipitates that are firmly embedded in the metal matrix. An important process during heat treatment is the so-called destabilisation of the austenite, as the martensitic transformation may occur only after this happens. During the cooling of the casting, the M_7_C_3_ carbide-precipitation process takes place at a slow rate; therefore, the austenite is supersaturated with alloying elements that stabilise it at an ambient temperature. On the other hand, longer heating at temperatures above 800 °C leads to the formation of the secondary M_7_C_3_ carbide precipitation; that is, the so-called austenite destabilisation. Depending on the cooling rate that follows the austenite destabilisation process, perlite, bainite, and martensite may be formed. The high proportion of residual austenite in the matrix is disadvantageous for the anti-abrasive properties. Its formation is favoured by too much chromium and a too-high annealing temperature of the cast iron before hardening.

## 2. Methodology

This research was carried out under industrial conditions at the foundry of Odlewnie Polskie S.A. in Starachowice, Poland. A medium-frequency induction furnace with a capacity of 120 kg was used; this was located at the Research and Development Centre for Foundry Components “OBRKO”. The molten metal was overheated to 1600 °C and transported in a 30 kg ladle to the casting mould pouring station. A set (each inoculant tested separately) of casting moulds was made of loose self-hardening sands in which a hot crack-resistance test was performed—the Althoff-Radtke test [22]. Samples for testing the impact toughness and abrasion resistance were also cut from the Althoff-Radtke test. Plates with dimensions of 100 × 100 × 30 mm were also cast for microstructure analyses, and a mould was fabricated for the dilatometric tests.

### 2.1. The Chemical Composition of Melts and Inoculants Used in the Experiments

The research used simple inoculants in the form of technically pure elements—Al, iron alloys (FeTi and FeB with different titanium contents), and complex inoculants that are currently part of the know-how (KH) of the authors of this article (KH 1–KH 7). Compound inoculants were made of technically pure elements at AGH’s Faculty of Foundry specially for the purpose of this work. The fabricated inoculants were crushed to grain size 1–2 mm and introduced into the molten metal. The chemical compositions of the alloys from chromium cast iron are presented in Table 1.

### 2.2. Toughness and Hardness of Chromium Cast Iron

The Charpy impact test, VEB WerkstoffprUfmaschinen (Germany) has been used. Unnotched samples were used. According to the PN-EN ISO 148-2010 standard, the test result is the ratio of the amount of work that corresponds to the energy that is used to break a sample to its cross-sectional area.

The Rockwell and Vickers methods were used to test the hardness. These test parameters are specified in the PN-EN-ISO 6508-1: 2002 standard. The Rockwell hardness tester 610 KABiD-PRESS (Poland) has been used. A diamond cone with an apex angle of 120° (for the C scale) was used as a penetrator.

The Vickers hardness tester HPO-250 VEB (Germany) was used.

### 2.3. Abrasive-Wear Resistance

The abrasive-wear resistance was tested on the MAN DNL463 Baumüller, Germany (the scheme of which is shown in [22]). The machine consisted of an electric motor that drove a high-carbon cast steel counter-sample disc. Above the disc, there was a loading arm to which previously prepared samples were placed. The device monitored the speed as well as the friction distance that was covered by the sample. The samples for the abrasive-wear test were 20 × 10 × 12 mm.

### 2.4. Optical and Scanning Microscope Analysis

A metallographic analysis was performed using a LEICA MEF-4M (Leica Microsystems, Wetzlar, Germany) optical microscope that was supported by an LEI-CA-Qwin automatic image analyser. An analysis of the microstructure and chemical composition was also performed on a JEOL 500 LV scanning microscope (JEOL Ltd., Tokyo, Japan).

### 2.5. Phase Composition Analysis Using XRD Radiation

The area of the application of XRD in the X-ray phase analysis was identifying the crystalline phases and determining the phase composition of the crystalline samples.

The SIEMENS X-ray diffractometer, model Kristalloflex 4H (Munich, Germany), was used. A CuKα = 1.54 Å lamp was used. CuKα radiation, vertical goniometer and counter registration of the angle and position of the reflex were used. The measurement range of the 2-theta angle was 5–135°, step: 0.05°. The time of counting a single impulse was assumed to be 1 s. The voltage was 20 kV and the current 30 mA. The experimental data were processed in order to remove the background calculation of the position, integral intensity and peak height. For this purpose, the XRayan computer program was used (software version 421). Phase identification consisted of comparing the obtained signals with reference signals contained in the database (International Centre for Diffraction Data PDF-2, 2018). Bragga range: 30–120°.

### 2.6. Dilatometric Tests and Heat Treatment

**Figure 4:**
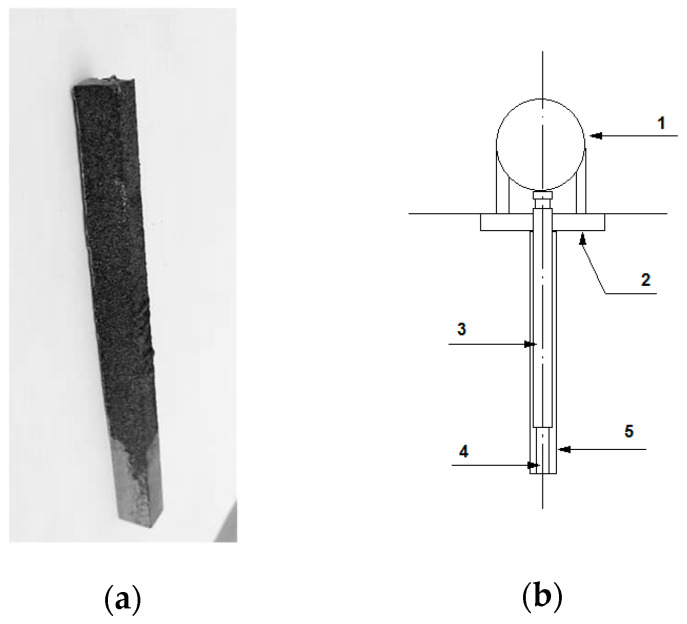
(**a**) Photograph of chromium cast iron ingot intended for dilatometric sample; (**b**) dilatometer construction diagram (1—Capacitive displacement sensor; 2—Fixing element; 3—Pusher, 4—Sample; 5—Protective quartz tube).

The dilatometer DI-105 PAN Instytut Mechaniczny (Gliwice, Poland) was used (Figure 4). Chromium cast iron test specimens were prepared under industrial conditions. The test castings in the shape of an ingot with the dimensions of 10 × 10 × 55 mm (Figure 4a) were made of chromium cast iron that was inoculated with 0.66% FeTi. In the research part, the samples were marked as follows: L—As cast; W—Annealing.

#### Heat-Treatment Parameters

The heat treatment was performed in a POK-72-type heat-treatment furnace (Poland).

Heat treatment of the tested cast iron consisted of heating at a rate of Vn = 10 °C/min at 640 °C for 60 min and cooling in air to the ambient temperature.

## 3. Hot Cracking-Resistance Tests (Althoff-Radtke Test)

The test of the resistance to hot cracking was performed on samples that were cast under industrial conditions for special forms of the Althoff-Radtke test. Chromium cast iron samples were tested, which were inoculated accordingly:
• KH 1;• 0.17% Al;• 0.17% FeB;• 0.25% FeTi;• KH 2;• 0.33% Al;• 0.33% FeB;• 0.50% FeTi;
• 0.50% Al;• 0.50% FeB;• 0.75% FeTi.

**Figure 5:**
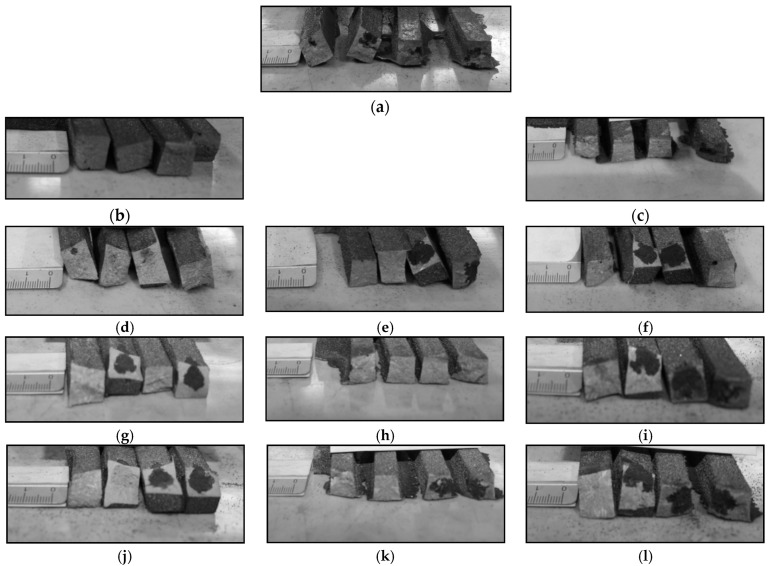
Results of Althoff-Radtke tests: (**a**) reference sample, inoculation; (**b**) KH 1; (**c**) KH 2; (**d**) 0.25% FeTi; (**e**) 0.50% FeTi; (**f**) 0.75% FeTi; (**g**) 0.17% Al; (**h**) 0.33% Al; (**i**) 0.50% Al; (**j**) 0.17% FeB; (**k**) 0.33% FeB; (**l**) 0.50% FeB for samples from left to right—50, 150, 250, and 350 mm. The dark areas indicate the local oxidation formed when the specimen fractured during solid-state shrinkage (hot cracking).

The casts were knocked out, and the arms were broken off. The hot-crack tendency was assessed based on the observation of the fractures in the castings. The photos of these fractures are shown in Figure 5.

The conducted research showed that the inoculation with KH 1 had the highest beneficial effect on the elimination of hot cracks. In this case, no shrinkage cavity appeared in any of the arms, nor were there any black areas that were indicative of defects. For each addition of an inoculant, a clear reduction in the defects can be seen as compared to the reference sample. Ferro-titanium inoculation also reduced the hot-cracking tendency. The smallest addition of FeTi (at an amount of 0.25%) had the most favourable effect. The 0.50% FeTi inoculation minimised the stresses in the shorter arms (50 and 150 mm), whereas dark areas (local oxidation formed when the specimen fractured during solid-state shrinkage) are visible in the longer arms (250 and 350 mm). The 0.75% FeTi inoculation only reduced the tendency to hot crack in the shortest leg (50 mm). Aluminium and FeB contributed the least to improving the resistance to hot cracking. The aluminium inoculation eliminated the shrinkage cavity and dark areas in the casting in only one case—the addition of 0.33% Al. On the other hand, a slight improvement was noticeable in the case of FeB as compared to the reference sample. It can be concluded from the conducted research that, for each inoculant, there was an optimal additive that influenced the elimination of hot cracks. For the inoculants KH, Al and FeTi the optimum addition was in the lower contents.

## 4. Toughness and Hardness Test

**Figure 6:**
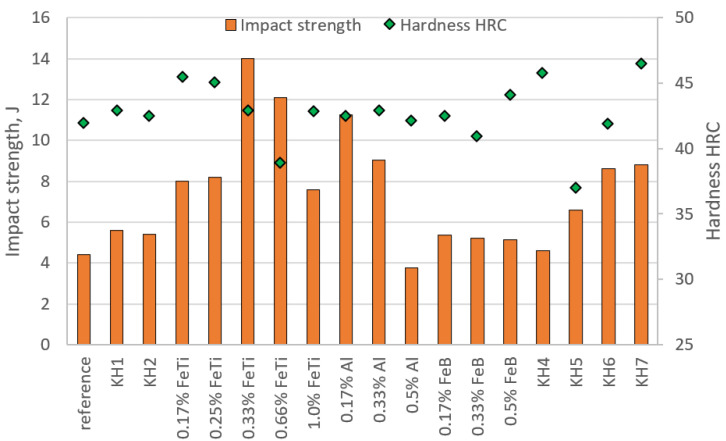
Results of toughness and hardness tests.

Two samples of each melt were subjected to impact tests; the averaged results of the impact toughness and HRC hardness measurements are presented in the diagram in Figure 6. As can be seen, there is a relationship between the hardness and the impact toughness. Those samples with good impact toughness have slightly lower hardness, and the samples with significantly improved hardness lost their impact toughness. In all cases, however, the hardness was too low for use under severe abrasive-wear conditions. Inoculations with KH 1 and KH 2 did not significantly improve the impact resistance nor the hardness, but the ferric titanium significantly improved the impact toughness. For the addition of 0.33% FeTi, the highest impact strength was achieved; this amounted to 14 J, which was an almost three-fold improvement when compared to the reference state. Further increases in the amounts of FeTi slightly reduced the impact toughness, so it can be predicted that further increases in FeTi will lower the impact toughness. The addition of aluminium improved the plasticity of the alloy (Figure 5h–j). The inoculation with the amounts of 0.17–0.33% Al increased the impact toughness more than two-fold, whereas the highest amount Al (0.5%) decreased it sharply. The last result was quite interesting because the fracture of the sample was plastic, but the test results were much lower—3.75 J. This may have been because, as an element, Al improves the plasticity of the alloy. However, a too-high addition reduces the impact toughness due to the appearance of Al_2_O_3_ at the primary austenite grain boundaries. FeB inoculation improved the impact toughness by approximately 1 J. Interesting results were also obtained for the compound inoculants. KH 4 significantly improved the hardness, but it did not significantly improve the impact toughness. In turn, KH 5 significantly lowered the hardness while improving the impact toughness. The combination of these two inoculants (KH 7) improved both the hardness and the impact strength (for the hardness, we achieved a two-fold increase). Therefore, we can conclude that, in such an inoculant, one component improves the hardness, whereas the addition of the second component increases the impact toughness. On the other hand, the use of the complex KH 6 inoculant (a physical mixture in the appropriate proportions of KH 5 and FeTi) worsened the impact toughness as compared to the inoculation with only 0.66% FeTi (while also improving the hardness). When comparing the other results, it seems more reasonable to apply the 0.33% FeTi inoculation since we can gain improved properties (impact strength and hardness) at a lower cost of production.

## 5. Testing Resistance to Abrasive Wear

**Figure 7:**
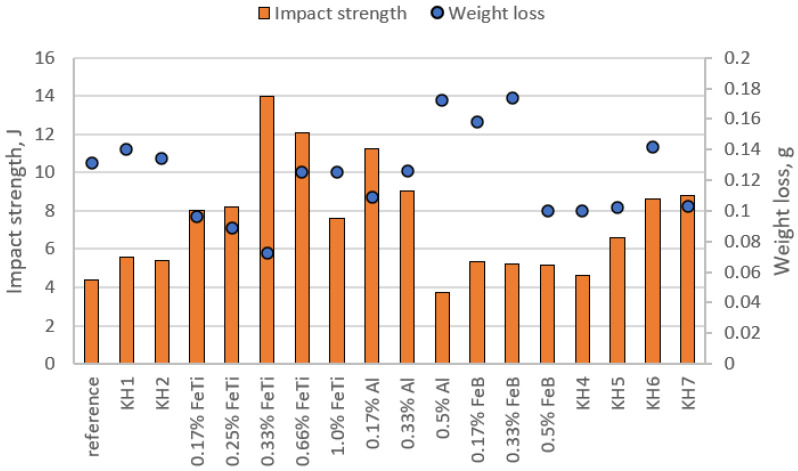
Results of tests of resistance to abrasive wear and impact toughness of chromium cast iron.

The samples that were used in the impact test were subjected to an abrasive wear-resistance test. The results of the abrasive wear-resistance tests are presented in Figure 7. The diagram shows the weight loss of each sample along a distance of 50 km on a MAN machine. The red line shows the weight loss of the reference sample; all the results above the red line were characterised by reduced resistance, and those below the line showed increased resistance to abrasion. Additionally, the results of the impact test for the individual samples are presented in the graph. A certain dependence can be noticed: for the inoculant for which the lowest weight loss was achieved (0.33% FeTi), the highest impact strength was achieved.

A similar dependence can be observed for the inoculant that had the least positive effect on the abrasion resistance—0.5% Al—where the weight loss was 0.172 g and the lowest impact strength was 3.75 J. As already mentioned, the best results were achieved for the addition of 0.33% FeTi; it can also be noticed that the addition of FeTi in other amounts had a positive effect on improving abrasion resistance. Those inoculations with the developed inoculants (KH 4, KH 5, and KH 7) also contributed significantly to reductions in weight loss. This is interesting, as the addition of some simple inoculants slightly reduced the abrasion resistance. Although the use of pure aluminium had a minimal effect on improving abrasion resistance, combinations of Al with some simple inoculants had very positive effects on reducing the weight loss in the samples.

Thus, the inoculation procedure greatly improved the wear resistance of high-chromium cast iron. It should also be emphasised that the tested high-chromium (hypoeutectic) cast iron was characterised by very good resistance to abrasive wear.

## 6. Analysis of Microstructure Using Optical Microscope

**Figure 8:**
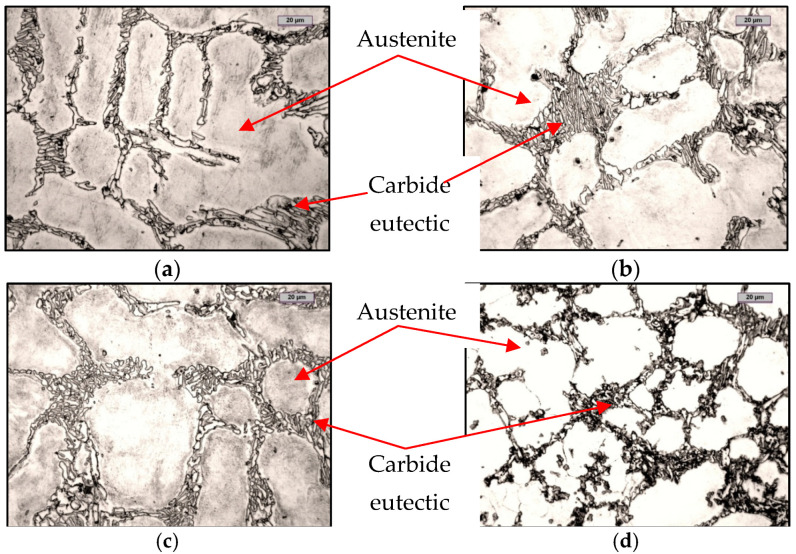
Microstructures of chromium cast iron samples: (**a**) reference; (**b**) inoculation of: 0.17% FeTi; (**c**) 0.33% FeTi; (**d**) 0.66% FeTi (magnification—500×).

Figure 8 shows the microstructures of the tested chromium cast iron castings. From these, it can be noted that the addition of FeTi changed the crystallisation from exogenous to endogenous. The reference sample had a clearly directed structure, whereas the addition of FeTi changed the crystallisation method to a volumetric one. FeTi inoculation also refined the microstructure. The microstructure shown above consisted of austenite dendrites (light areas) and carbides.

## 7. Analysis of Microstructure Using Scanning Microscope

### 7.1. Reference Cast Iron

**Figure 9:**
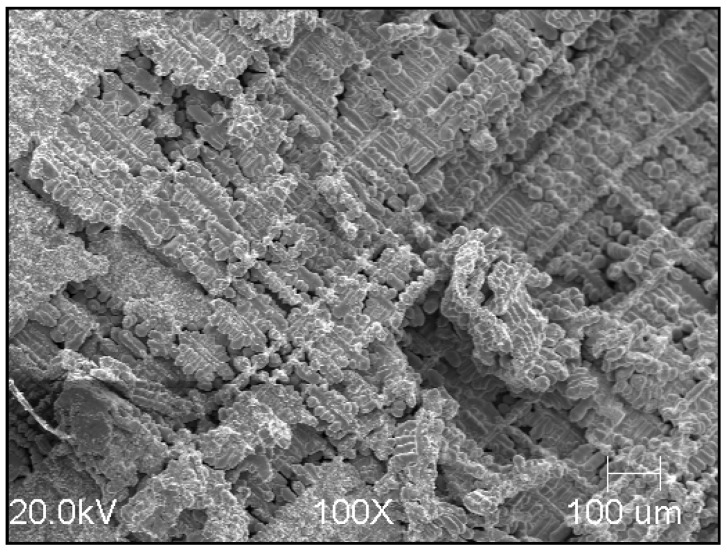
Fracture of reference sample (SEM; magnification—100×).

**Figure 10:**
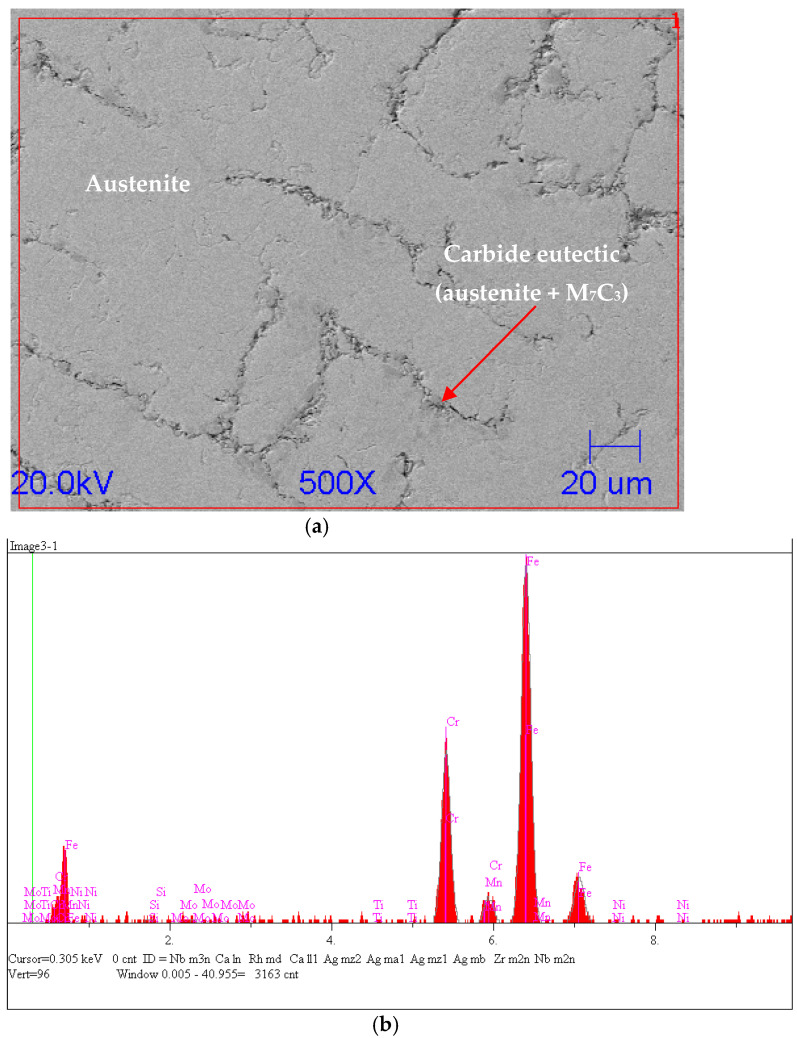
(**a**) Scanning microscope analysis of reference sample; (**b**) qualitative EDS analysis from area.

For the reference sample, photos of the fracture (Figure 9) and the polished surface (Figure 10a) were taken.

It can be seen that the microstructure of the casting consisted mostly of austenite dendrites. Figure 10b shows the EDS analysis from the surface of the reference sample.

**Figure 11:**
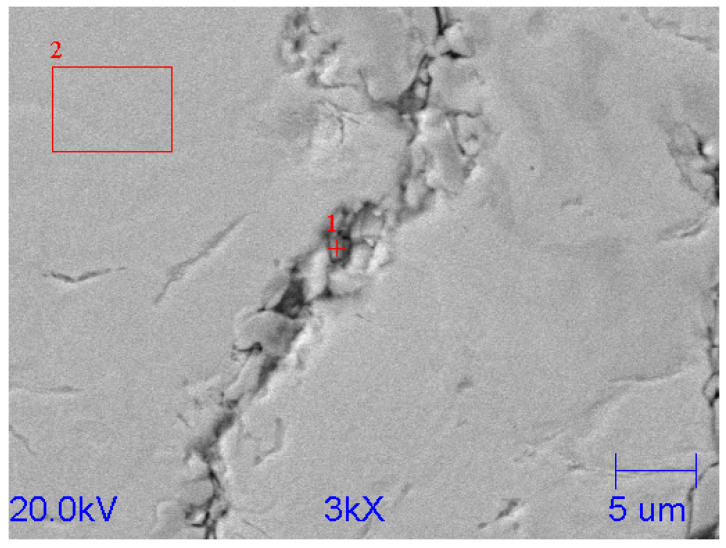
SEM microstructure of reference chromium cast iron.

In order to determine the type of carbide, a point analysis was performed; in addition, the chemical composition of the metal matrix was determined. The location of the analysis is shown in Figure 11.

**Figure 12:**
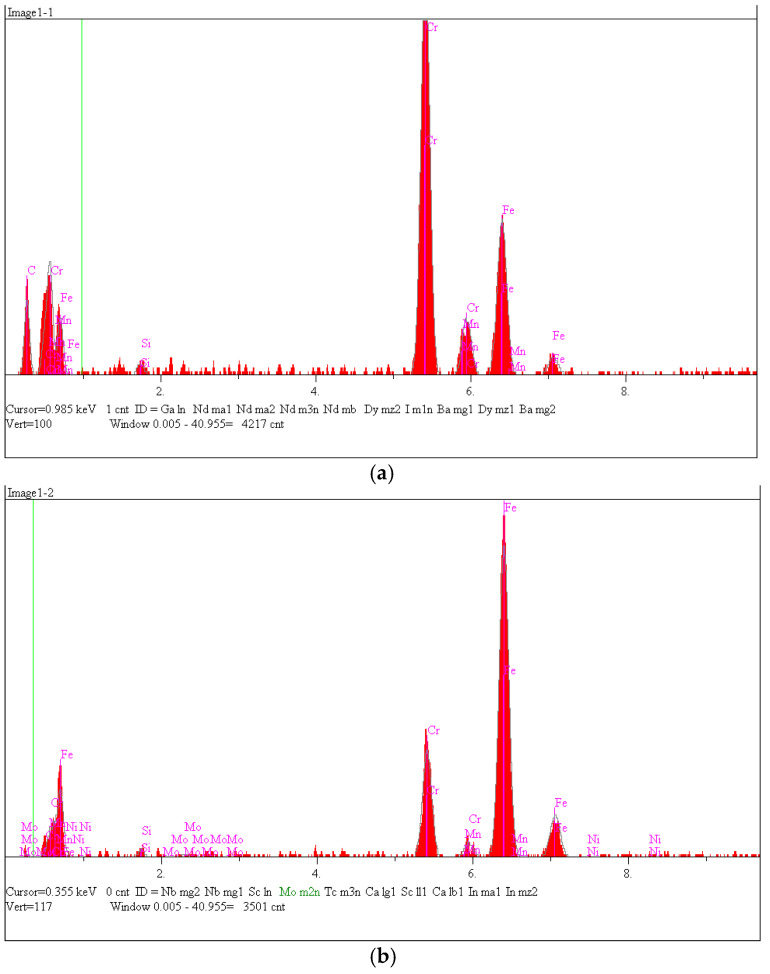
Scanning microscope analysis of reference sample: (**a**) qualitative EDS analyses from Point 1 (carbide); (**b**) qualitative EDS analyses from Point 2 (matrix).

An EDS analysis (shown in Figure 12, Table 2 and Table 3) showed that the sample consisted of a matrix with mainly 82% at. Fe, 14% at. Cr, and chromium carbides. The shape of the carbide and the ratio of the chromium and carbon indicated that this was M_7_C_3_.

### 7.2. Analysis of Structure of FeTi-Inoculated Chromium Cast Iron

**Figure 13:**
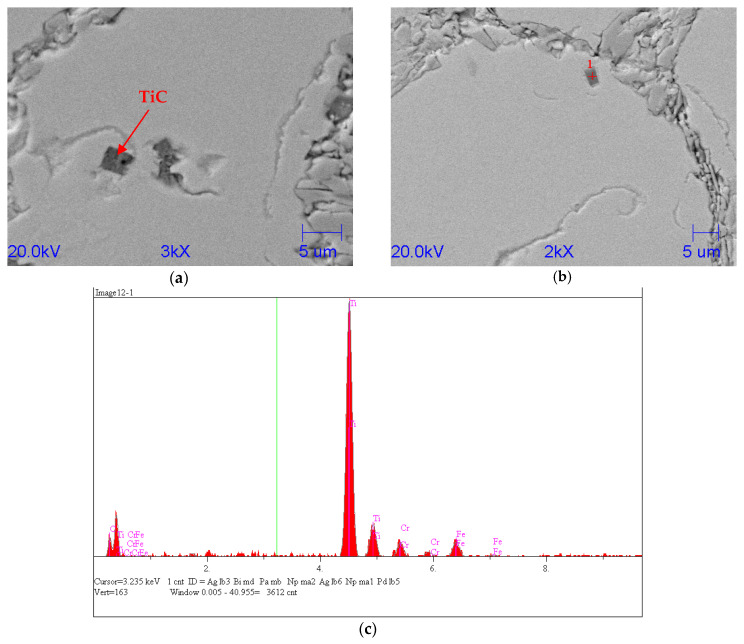
Scanning microscope analysis of inoculated sample with 0.33% FeTi: (**a**), (**b**) SEM; (**c**) qualitative EDS analyses, respectively, from Point 1.

The FeTi inoculation led to the appearance of a small amount of TiC with small dimensions in the cast iron’s structure. Figure 13 shows the exemplary microstructure of chromium cast iron with TiC precipitates. Figure 13 and Table 4 shows SEM pictures and an EDS analysis of the microstructure of an inoculated FeTi sample. In Figure 13b–d, the analysis results of the chromium carbide are shown, and Figure 13a shows the titanium carbide. This shows the characteristic shape of a cuboid that is taken by the titanium carbide. Here too, the structure of the titanium carbide is linked to additional nitrogen atoms.

## 8. Phase Composition Analysis Using XRD Technique

**Figure 14:**
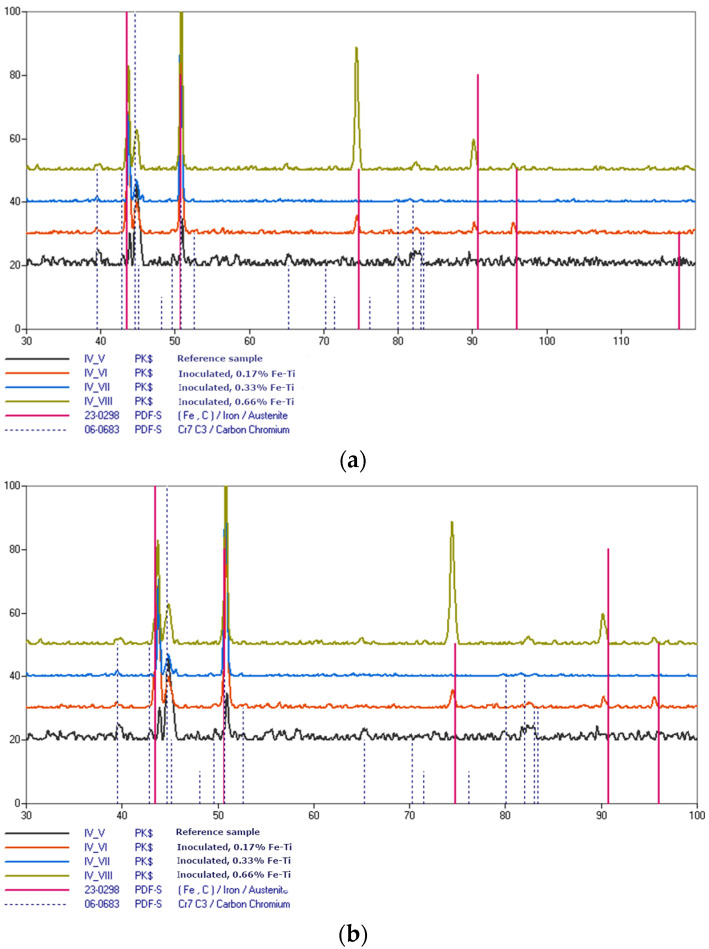

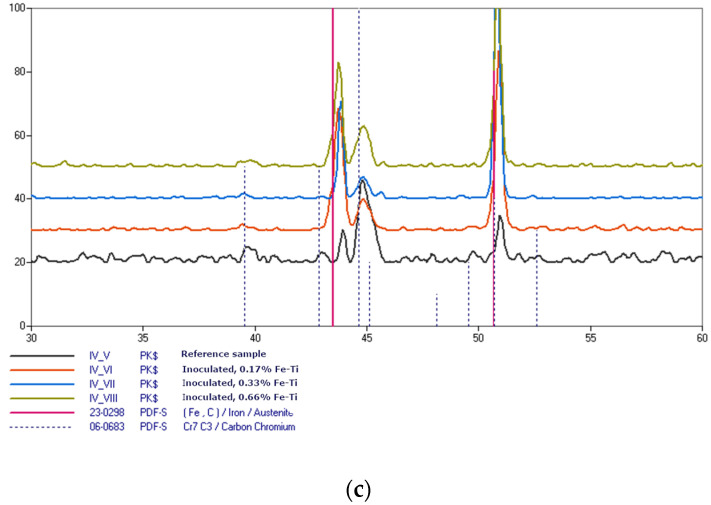
X-ray diffraction patterns of analysed samples of hypoeutectic high-chromium cast iron that was inoculated with FeTi in following ranges: (**a**) 30–120°; (**b**) 30–100°; (**c**) 30–60°.

The X-ray diffraction method (XRD) was utilized to phase analysis. Only the FeTi inoculated samples were subjected to this test. The same set of samples were tested for analysis on the scanning microscope; i.e., the reference sample as well as the samples that were inoculated with 0.17, 0.33, and 0.66% FeTi. Figure 14 shows the obtained diffractograms for the samples with the hypoeutectic compositions for different ranges of angles.

The conducted research shows that the microstructure of the castings consisted of austenite grains and Cr_7_C_3_-type chromium carbides. As the amount of titanium increased, the diffraction patterns of the subsequent samples were smoother; therefore, inoculation with titanium reduced the noise (which may mean a more ordered structure). An interesting case was the inoculation of 0.33% FeTi (blue line); the lines were smoothed, the sizes of the peaks were reduced to within a range of 60–120°, and increased to within a range of 30–60°. As already mentioned, a smoother diffractogram may mean a sample with a more ordered structure. Among the tested samples, the sample that was inoculated with 0.33% FeTi was characterised by the smoothest graph. The best strength properties were obtained for this inoculant addition; therefore, this may mean that the structure after inoculation had the best influence on the strength properties (with the features of the ordered grains evenly distributed in the structure). Figure 14 shows that the peak for the austenite increased although the peak for the chromium carbide decreased within a range of 42–46° with increasing additions of titanium; this might seem surprising. However, the peak for the chromium carbide coincided with the peak for the austenite within a range of 50–55°, so it should be assumed that the increased peak intensity for the subsequent titanium doses referred to the chromium carbide M_7_C_3_ in this case and not to the austenite grains. The noise may have also come from a certain orientation of the structure that resulted from the sampling methodology that was obtained from the Althoff-Radtke sample (i.e., low-mass castings). The conducted research did not identify titanium carbides in the phase composition. The results are interesting because the scanning microscope studies clearly showed the presence of this carbide for the inoculated samples with both 0.33 and 0.66% FeTi. The inoculants used do not affect the morphology of the chromium carbide formation.

## 9. Heat Treatment

**Figure 15:**
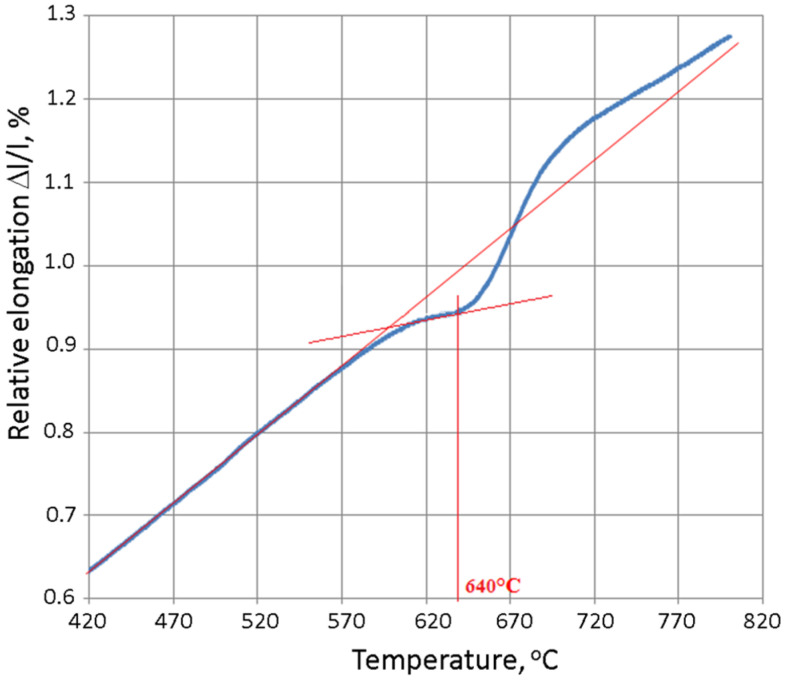
Relative elongation (blue line) of tested cast iron as function of temperature for Vn = 1 °C/min. Red lines - tangents determining the kinetics of austenite destabilization.

Figure 15 shows the change in the relative elongation of the tested cast iron as a function of temperature at a heating rate of Vn = 1 °C/min. A preliminary analysis of the above dependence allows us to determine the annealing temperature that leads to austenite destabilisation. The deviation from the linear dependence of the relative elongation during the heating of cast iron is the result of the separation of the secondary carbides from the austenite and, consequently, the latter’s destabilisation; this takes place at a temperature of 640 °C.

**Figure 16:**
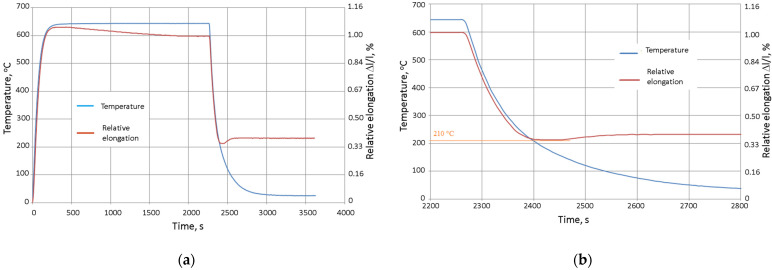
(**a**) Relative elongation of tested cast iron during heat treatment; (**b**) change of relative elongation of tested cast iron during cooling.

Figure 16 shows the dimensional changes that resulted from the heating, annealing (at 640 °C), and cooling of the cast iron. The decreasing value of the relative elongation during the annealing was the result of the separation of the secondary carbides from the austenite; this led to its partial destabilisation (Figure 16a). During the cooling from a temperature of about 210 °C, the effect of increasing the length of the sample was visible; this was related to the diffusion-free transformation of the austenite into martensite (Figure 16b).

### Metallographic Tests and Quantitative Analysis of Samples after Heat Treatment

**Figure 17:**
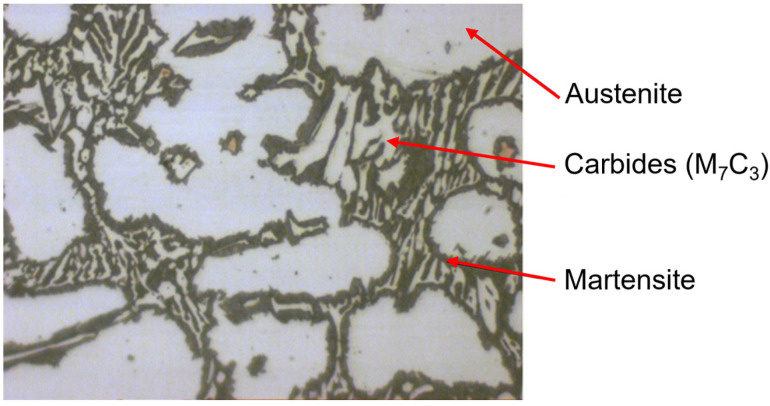
Microstructure of chromium cast iron after heat treatment and etched with solution of nitric acid in ethanol, magnification—1000×.

Before the heat treatment, microscopic images showed light austenite grains and a darker carbide eutectic that consisted of a mixture of austenite and M_7_C_3_ alloy carbides. Images of the inoculated 0.66% FeTi cast iron after heat treatment are shown in Figure 17. The microstructure of the tested cast iron that was revealed as a result of etching with a 4% solution of nitric acid in ethanol suggests that only the part of the austenite that was located in the vicinity of the eutectic carbides was destabilised and transformed into martensite. The volume fraction of the martensite after the destabilising annealing was VM = 12%.

#### 9.1.1. Abrasive-Wear Resistance of Samples after Heat Treatment

**Figure 18:**
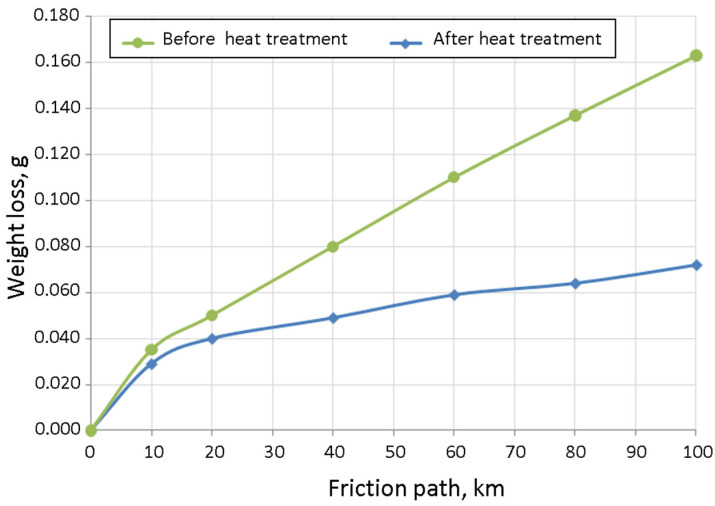
Amount of abrasive wear for cast iron before and after heat treatment depending on friction path travelled.

The abrasion-resistance test was performed before and after the heat treatment. With a unit pressure of P = 0.2 MPa, the weight loss was measured after the 0, 10, 20, 30, 40, 60, 80, and 100 km of distances that were travelled on the friction path on the MAN device. The wear mechanism of the test sample was micro-cutting. The results of this study are shown in Figure 18.

The abrasive-wear effect during the initial range of 0 to 10 km of the friction path did not show any significant differences. Within a range of 10 to 100 km, however, the differences in the abrasion were clearly visible. At 100 km, the sample after annealing was less than half as abrasive as was the other sample. This improvement was the result of the appearance of martensite in the microstructure and the increase in the volume fraction of the carbide phase.

#### 9.1.2. Impact Strength and Hardness of Chromium Cast Iron after Heat Treatment

The results of the impact toughness and hardness tests of the chromium cast iron that was inoculated with 0.66% FeTi after heat treatment are presented in Table 5.

The designed heat treatment more than doubled the resistance to abrasive wear, and the hardness value increased by nearly 50%; however, the impact toughness was cut in half.

## 10. Discussion and Conclusions

The conscious shaping of the structures and improvements of selected properties of chromium cast iron castings was aimed at optimising the casting technology in order to obtain a product that met technical requirements at the same time as minimising costs. The process that leads to obtaining this type of casting is related to inoculation and heat treatment; however, it seems that carrying out a complete heat treatment for inoculated cast iron may be disadvantageous due to the precipitation of the residual austenite. Thus, the targeted heat treatment that is proposed in this paper can serve this purpose. The parameters of the applied heat treatment were determined on the basis of dilatometric tests. The deviation that is visible on a dilatogram at a temperature of about 600 °C is the result of the separation of the secondary carbides from the austenite, which leads to the latter’s destabilisation and partial transformation into martensite. The effect of a diffusionless martensitic transformation was illustrated by the increase in the length of the sample during cooling from a temperature of about 210 °C. The increase in abrasion resistance was mainly due to the appearance of martensite (the appearance of secondary carbides could have also contributed to this, but their quantity and, therefore, their effect have not been determined). The microstructure of the tested cast iron (soft matrix, hard carbides) is particularly desirable in the case of alloys that work with lubrication. The micro-cavities that are formed by wiping the softer phase constitute natural reservoirs of grease, which reduces abrasive wear. The changes in the microstructure that resulted from the applied heat treatment had a negative impact on the impact toughness, causing it to drop more than two-fold. This was due to the appearance of martensite (which has a lower toughness than austenite). The structure in which part of austenite located near the carbides is de-stabilized and transformed into martensite is also important. Too great a drop in impact toughness often disqualifies a material (even with high abrasion resistance) due to the development of cracks during its operation. However, the achieved values are acceptable for selected chromium iron castings in the cases of the presented tests. The conducted research and its subsequent analysis allowed for the following conclusions:

- Inoculation treatment reduces the tendency of chromium cast iron castings toward hot cracking.
- Inoculation treatment in most of the tested inoculants had a neutral or slightly increasing hardness effect. In the case of using KH 5 (CuBi), KH 6 (CuBi-FeTi) and 0.66% FeTi, a visible reduction of HRC hardness was observed.
- The use of inoculants (except 0.5% Al) increases the toughness. The use of 0.33% FeTi increased it more than threefold.
- Annealing the tested chromium cast iron at a temperature of 640 °C caused a partial destabilisation of the austenite (volume fraction of martensite = 12%).
- The heat treatment increased the tested chromium cast iron hardness by 54%.

## Figures and Tables

**Table 1:** Chemical composition of chromium cast iron and the inoculants used.

**Chemical Composition of Reference Cast Iron, wt.%**									
	**C**	**Si**	**Mn**	**P**	**S**	**Cr**	**Ni**	**Al**	**Ti**
Reference iron	1.996	0.538	0.454	0.02	0.013	21.796	0.16	0.0029	0.0096
**Chemical Composition After Adding Inoculants** **, wt.%**									
**Inoculant**	**C**	**Si**	**Mn**	**P**	**S**	**Cr**	**Ni**	**Al**	**Ti**
KH 1	1.754	0.609	0.429	0.021	0.0133	22.495	0.195	0.0034	0.0065
KH 2	1.818	0.618	0.448	0.021	0.012	22.586	0.194	0.0037	0.0066
0.17% FeTi	1.78	0.79	0.47	0.017	0.01	21.22	0.18	0.0085	0.0599
0.25% FeTi	1.962	0.535	0.362	0.019	0.0119	21.632	0.161	0.0099	0.129
0.33% FeTi	1.77	0.77	0.42	0.019	0.012	21.11	0.19	0.0102	0.0882
0.66% FeTi	1.67	0.77	0.43	0.017	0.01	21.06	0.18	0.0158	0.204
1.0% FeTi	1.878	0.596	0.429	0.015	0.0204	22.707	0.187	0.0418	0.6003
0.17% Al	1.865	0.604	0.364	0.019	0.0122	22.864	0.191	0.0785	0.0072
0.33% Al	1.864	0.59	0.405	0.018	0.0104	22.656	0.192	0.1921	0.0085
0.5% Al	1.768	0.593	0.416	0.018	0.011	22.833	0.194	0.2393	0.0096
0.17% FeB	1.836	0.661	0.43	0.02	0.0151	23.189	0.192	0.0168	0.0096
0.33% FeB	1.85	0.627	0.413	0.022	0.0154	22.96	0.196	0.0065	0.0077
0.5% FeB	1.837	0.608	0.406	0.021	0.0156	23.257	0.196	0.0034	0.0070
KH 4	1.96	0.41	0.35	0.017	0.012	21.71	0.21	0.3063	0.0022
KH 5	1.73	0.3	0.3	0.015	0.01	21.04	1.37	0.0055	0.0033
KH 6	1.47	0.3	0.3	0.015	0.01	21.02	1.37	0.0055	0.1521
KH 7	1.98	0.37	0.31	0.017	0.011	22.07	0.22	0.1584	0.0021

KH 1—Bi, KH 2—Bi, KH 4—AlBi (alloy), KH 5—CuBi (alloy), KH 6—CuBi + FeTi (physical mixture of alloys), KH 7—CuBi + AlBi (physical mixture of alloys).

**Table 2:** Quantitative EDS analyses analyses, respectively, from Point 1 (carbide).

	C	Si	Cr	Mn	Fe
Atomic, %	68.076	0.522	19.947	0.210	11.245
Conc, wt %	32.588	0.584	41.338	0.460	25.030

**Table 3:** Quantitative EDS analyses, respectively, from Point 2 (matrix).

	C	Si	Cr	Mn	Fe	Ni	Mo
Atomic, %	9.928	1.388	14.114	0.0	74.122	0.205	0.244
Conc, wt %	2.353	0.769	14.483	0.0	81.695	0.238	0.461

**Table 4:** Quantitative EDS analyses from Point 1 (TiC).

	C	N	Si	Ti	Cr	Mn	Fe	Co	Ni	Mo
Atomic, %	31.015	37.611	0.315	25.708	1.981	0.543	2.786	0.0	0.019	0.021
Conc, wt %	15.328	21.676	0.364	50.633	4.239	1.228	6.402	0.0	0.047	0.083

**Table 5:** Impact strength and hardness of chromium cast iron inoculated with addition of 0.66% FeTi after heat treatment.

Impact Strength, J		Hardness HV	
Heat Treatment			
before	after	before	after
14.0	5.4	383	594
13.2	5.5	387	594
9.8	5.6	380	590
Average = 1.3	Average = 5.5	Average = 383	Average = 593

## Data Availability

Not applicable.
